# Ethics

**Published:** 1904-05-15

**Authors:** F. W. Harnden

**Affiliations:** San Francisco


### ETHICS.

BY DR. F. W. HARNDEN, SAN FRANCISCO.

It is not the purpose of this paper to deal with the question of ethics in its broad application to life in general, nor to collect for your edification choice quotations from Plato, Machiavelli, Herbert Spencer, Emerson or any others of the great teachers of moral philosophy, but to advance a few simple commonplace ideas on the subject of ethics as they should control us in our every-day experiences as professional men.

When we learned that it was best to train men carefully for special work along any special line, we began to see the necessity for rules and regulations for the protection of both the individual members of the craft and of the craft itself.

When the craft grew into a profession, then the "rules and regulations" became a code of "ethics."

When we subscribe to and live up to a code of ethics, we are professional men; and when we digress from or refuse to be governed by such a code, we become mere craftsmen.

Any profession which is governed by a code of ethics makes it obligatory that the individual members shall have been properly qualified to enter it, and having entered shall so conduct themselves that the best interests of the profession shall be advanced. The formation and support of dental societies—alumni, city, state, national and international; the existence of technic and pedagogic institutes, and the maintenance of the fraternal organizations are all for the one object of the betterment of the profession.

When an individual, in the hope of personal gain, throws over his allegiance to his profession, he works a double injury—to himself and to his profession. It is folly to assume that an unethical practitioner is necessarily dishonest, but the tendency of such a course is distinctly demoralizing. When such a man resorts to open advertising, he claims that he performs as good a service as a non-advertising man for a *smaller* fee, or that he does *better* work at the same fee, or that he has a *private system* that revolutionizes. In any case, it is the individual and not the profession at large that he seeks to benefit, and in so doing he breaks down the helpful bond of good fellowship and professional fraternity.

But it is not the open advertiser who puts his name or an assumed name in the daily paper, with price list and guarantee attached, who is the greatest offender against a code of ethics, but rather he who while still enjoying the benefits of his society or societies deliberately violates the very provisions he is pledged to support. Such a man is distinctly dishonest, for he tries to appear what he is not, and it is clearly the duty of such societies to rid themselves of one who so forfeits his right to membership.

Perhaps the most flagrant abuse against a code of ethics is the use of the newspapers to exploit individual achievement. The reporting of but one or two clinics from among a large number, with fulsome praise of the favored clinicians; the reference to one man's work as being pre-eminent among many; the printing of portraits from either cuts or photographs, with biographical sketches of the resident or visiting practitioner; the announcement of the advent of a "*specialist*" in any particular branch of professional science; the description of a device or method as being one man's improvement over those in general use; these and many other schemes have been resorted to by those who wish to secure for themselves the recognition of the reading public, without suffering the loss of caste that comes from open advertising.

The excuse often made by the beneficiary of such newspaper notoriety is that personally he knew nothing of it until the "harm had been done," and that the exuberant interest of a friendly reporter or editor accounts for the article in question. This may be true in rare cases, but the general proposition that "we get nothing for nothing in this world" would seem to apply here. No amount of friendliness on the part of a weekly paper will keep a notice of a man's removal to new offices running in its columns for years after he has moved, and his clientele has forgotten the fact that he was ever in another building.

But there are other ways of reaching the dear public beside the newspapers.

The average person who receives through the mail a pamphlet purporting to be a paper read before a scientific society, and containing methods of treatment of diseased conditions of the human body, smiles a knowing smile when the name and address of the writer stares him in the face. His gratitude for the knowledge of the boon to suffering humanity held out to him is lessened considerably when he finds that the enterprising pamphleteer is unknown to him, but apparently wants to be known.

The public is being educated, and very rapidly, too, as to what constitutes non-ethical practices; and an effort by any individual to attract its attention is often met with the remark "he must be in need of patients." We are either ethical practitioners or we are not. There is no half way station at which to stand.

When we as individuals do all in our power to promote the interests and elevate the standard and dignity of our profession as a profession, we are ethical members; when we seek only personal profit or glory, we are *not* ethical members.

The same thing holds good with societies as with individuals. The jealousy—petty or great—existing between different fraternities, societies, colleges and faculties, is much to be deplored.

Theoretically, the fundamental purpose of all these organizations is the advancement of the profession in usefulness and dignity. In reality there is considerable effort expended in trying to belittle the work of each other. An honest zeal in making our fraternity, society or college the best we can make it is certainly a laudable ambition, and need not hinder in any way the efforts of the other fraternity, society or college. There is room for all when all are working for the same end—that of maintaining our profession at such a status that it will be considered an honor and a privilege to be one of its members.—*Pacific Gazette.*

